# Spinal Cord Perfusion Pressure Correlates with Anal Sphincter Function in a Cohort of Patients with Acute, Severe Traumatic Spinal Cord Injuries

**DOI:** 10.1007/s12028-021-01232-1

**Published:** 2021-06-07

**Authors:** Florence R. A. Hogg, Siobhan Kearney, Mathew J. Gallagher, Argyro Zoumprouli, Marios C. Papadopoulos, Samira Saadoun

**Affiliations:** 1 Academic Neurosurgery Unit, MCS Institute, St. George’s, University of London, London, SW17 0RE UK; 2 Neuroanaesthesia Department and Neuro Intensive Care Unit, St. George’s Hospital, London, UK

**Keywords:** Anal sphincter, Anal manometry, Blood pressure, Spinal cord injury, Spinal cord perfusion pressure

## Abstract

**Background:**

Acute, severe traumatic spinal cord injury often causes fecal incontinence. Currently, there are no treatments to improve anal function after traumatic spinal cord injury. Our study aims to determine whether, after traumatic spinal cord injury, anal function can be improved by interventions in the neuro-intensive care unit to alter the spinal cord perfusion pressure at the injury site.

**Methods:**

We recruited a cohort of patients with acute, severe traumatic spinal cord injuries (American Spinal Injury Association Impairment Scale grades A–C). They underwent surgical fixation within 72 h of the injury and insertion of an intrathecal pressure probe at the injury site to monitor intraspinal pressure and compute spinal cord perfusion pressure as mean arterial pressure minus intraspinal pressure. Injury-site monitoring was performed at the neuro-intensive care unit for up to a week after injury. During monitoring, anorectal manometry was also conducted over a range of spinal cord perfusion pressures.

**Results:**

Data were collected from 14 patients with consecutive traumatic spinal cord injury aged 22–67 years. The mean resting anal pressure was 44 cmH_2_O, which is considerably lower than the average for healthy patients, previously reported at 99 cmH_2_O. Mean resting anal pressure versus spinal cord perfusion pressure had an inverted U-shaped relation (*Ȓ*^2^ = 0.82), with the highest resting anal pressures being at a spinal cord perfusion pressure of approximately 100 mmHg. The recto-anal inhibitory reflex (transient relaxation of the internal anal sphincter during rectal distension), which is important for maintaining fecal continence, was present in 90% of attempts at high (90 mmHg) spinal cord perfusion pressure versus 70% of attempts at low (60 mmHg) spinal cord perfusion pressure (*P* < 0.05). During cough, the rise in anal pressure from baseline was 51 cmH_2_O at high (86 mmHg) spinal cord perfusion pressure versus 37 cmH_2_O at low (62 mmHg) spinal cord perfusion pressure (*P* < 0.0001). During anal squeeze, higher spinal cord perfusion pressure was associated with longer endurance time and spinal cord perfusion pressure of 70–90 mmHg was associated with stronger squeeze. There were no complications associated with anorectal manometry.

**Conclusions:**

Our data indicate that spinal cord injury causes severe disruption of anal sphincter function. Several key components of anal continence (resting anal pressure, recto-anal inhibitory reflex, and anal pressure during cough and squeeze) markedly improve at higher spinal cord perfusion pressure. Maintaining too high of spinal cord perfusion pressure may worsen anal continence.

**Supplementary Information:**

The online version contains supplementary material available at 10.1007/s12028-021-01232-1.

## Introduction

Traumatic spinal cord injury (TSCI) is a devastating event resulting in life-long disability, including limb weakness, bladder and bowel incontinence, sexual dysfunction, autonomic dysfunction, and, in cervical trauma, impaired breathing. TSCI affects approximately 180,000 people globally each year [1]. A major problem after TSCI is anal sphincter dysfunction from impaired control of the voluntary sphincter, impaired control of pelvic floor muscles, and impaired autonomic activity. Bowel function is ranked on par with limb weakness by patients with TSCI when prioritizing recovery goals [2]. Bowel dysfunction affects approximately 80% of patients with TSCI and is often ranked as more problematic than urinary incontinence or sexual dysfunction [3]. Fecal incontinence is common and often unpredictable. Constipation, diarrhea, and nausea are also commonly encountered, and most patients take drugs and require manual stimulation to defecate [4]. Such bowel regimens may consume a considerable amount of time.

Anal function may be assessed with anorectal manometry (ARM), which measures anal pressure (AP), rectal sensation, and the neural reflexes needed for normal defecation [5]. ARM allows the study of the sphincter when straining, resting, and squeezing to identify incontinence issues linked to the inability of the muscles to contract correctly and difficulties in defecating due to the incomplete opening and releasing of the sphincter muscles.

To improve the management of patients with TSCI in the neuro-intensive care unit (neuro-ICU), our group has developed monitoring from the injury site [6–9]. We monitor intraspinal pressure (ISP) and calculate spinal cord perfusion pressure (SCPP) as the mean arterial pressure (MAP) minus ISP. Monitoring is safe [10] and analogous to intracranial pressure and cerebral perfusion pressure monitoring for brain injury [11]. ISP and SCPP are clinically important parameters that correlate with injury-site metabolism [7, 12], neurological status [6, 13], urinary function [14], and long-term outcome [15].

Our objective was to test the effect of TSCI on anal function in the acute care setting. We performed ARM while also monitoring ISP and SCPP in the first 10 days after TSCI. We hypothesized that AP, change in AP (ΔAP) during squeeze or cough, recto-anal inhibitory reflex (RAIR), and rectal sensation are all influenced by ISP and SCPP. We also hypothesized that interventions to normalize these parameters could improve anal sphincter function.

## Methods

### Institutional Research Board Approvals

Approvals for the Injured Spinal Cord Pressure Evaluation (ISCoPE) study, including the consent form and patient information sheet, were obtained by the St. George’s Joint Research Office and the National Research Ethics Service–Camberwell St. Giles Ethics Committee (No. 10/H0807/23). ISCoPE is registered at www.ClinicalTrials.gov under identifier NCT02721615.

### Inclusion and Exclusion Criteria

We recruited patients with consecutive TSCI who were enrolled in the ISCoPE trial in the period of June 2018–August 2019. Inclusion criteria for ISCoPE were severe TSCI (defined as American Spinal Injury Association Impairment Scale [AIS] grades A–C), age 18–70 years, and surgery performed within 72 h of TSCI. Exclusion criteria were major comorbidities, the inability to obtain consent, and penetrating TSCI.

### Clinical Examination and Imaging

All patients were admitted to the neurosurgical unit at St. George’s Hospital and underwent an International Standards for Neurological Classification of Spinal Cord Injury AIS assessment by a neurosurgical resident trained in AIS, which was repeated at discharge and at the follow-up outpatient clinic. All patients had computed tomography and magnetic resonance imaging of the spine before surgery and within 4 weeks of surgery.

### Bowel Function at Follow-Up

In clinic, functional and quality of life measures were completed using two standard scales, the Spinal Cord Independence Measure III (SCIM III) bowel score [16] and the Neurogenic Bowel Dysfunction (NBD) score [17]. The SCIM III bowel scores are the following: 0, irregular timing or very low frequency (less than once in 3 days) of bowel movements; 5, regular timing, but requires assistance (e.g., for applying suppositories), and rare accidents (less than twice a month); 8, regular bowel movements, without assistance, and rare accidents (less than twice a month); and 10, regular bowel movements, without assistance, and no accidents. In the NBD score, bowel dysfunction is classified as the following: 0–6, very minor; 7–9, minor; 10–13, moderate; and 14 or higher, severe.

### Spinal Surgery

Surgical decompression, including laminectomies and spinal instrumentation, was performed based on patient requirements and surgeon preference. Lateral mass screws (cervical spine) or pedicle screws (thoracolumbar spine) were inserted above and below the fracture and were linked to rods secured with blockers using Oasys or Xia 3 (Stryker, Newbury, UK). Postoperatively, all patients were managed in the neuro-ICU.

### Probe Insertion

During posterior surgery, a pressure probe (Codman Microsensor Transducer; DePuy Synthes, Leeds, UK) was inserted through the skin into the wound cavity. Using a microscope, the dura and arachnoid were opened one level below the injury. The pressure probe was inserted intradurally, with the tip placed at the site of maximal cord swelling. The dural opening was sutured. This technique is described in detail elsewhere [6, 9, 10, 12, 13, 15, 18, 19]. ISP measured this way differs from intrathecal pressure measured above or below the injury site because the injured cord is compressed against the dura, thus compartmentalizing the intrathecal space [15, 20–22].

### ISP and Arterial Blood Pressure Monitoring

**Fig. 1:**
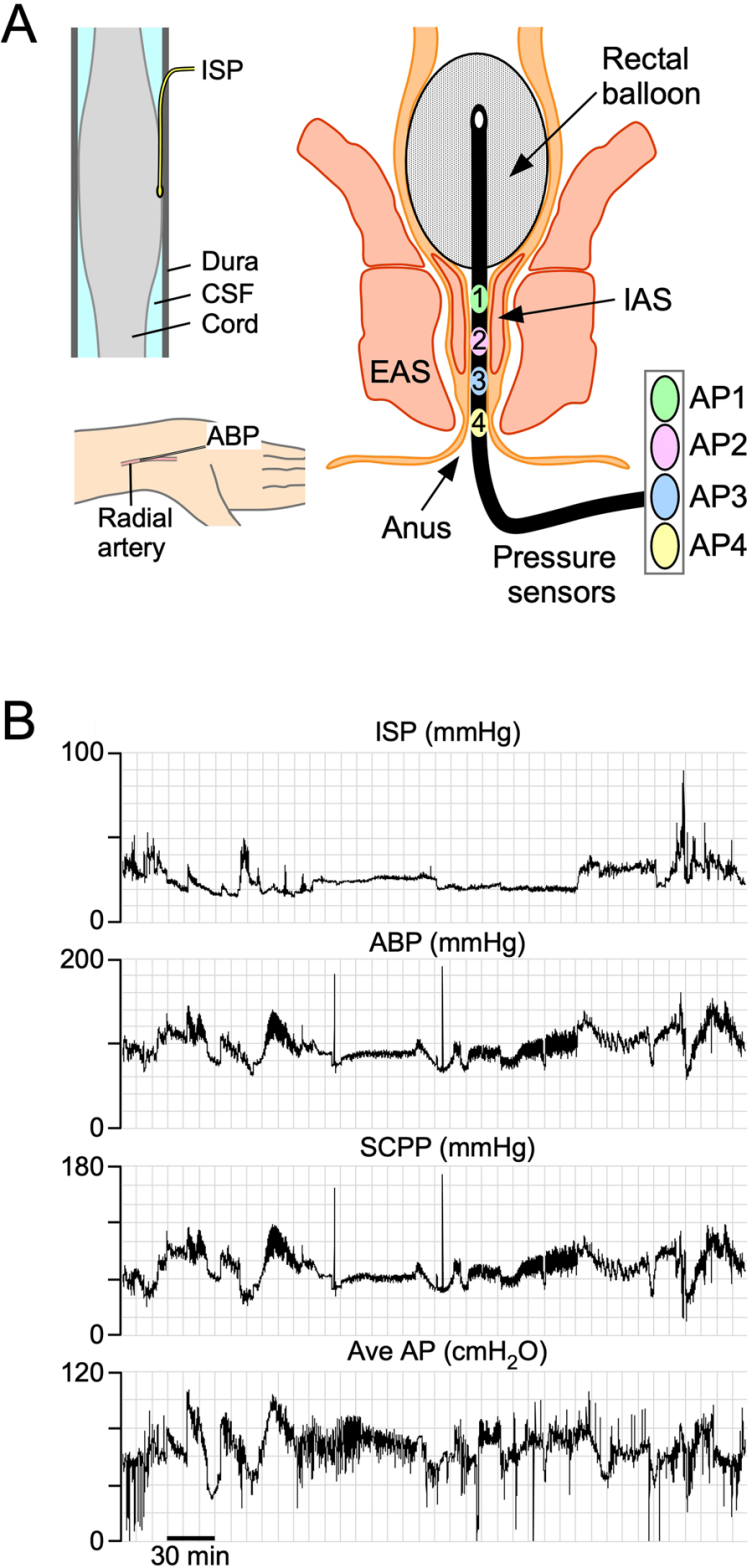
Setup for monitoring. **a** Intradural pressure probe monitors ISP (top left). Radial artery catheter monitors ABP, used to compute SCPP as MAP minus ISP (bottom left). Anal probe monitors from four pressure sensors to compute average AP and maximum AP (right). Rectal balloon used to assess sensation. **b** Examples of signals monitored simultaneously, including ISP, ABP, SCPP, and average AP. ABP arterial blood pressure, AP anal pressure, CSF cerebro spinal fluid, EAS extrenal anal sphincter, IAS internal anal sphincter, ISP intraspinal pressure, MAP mean arterial pressure, SCPP spinal cord perfusion pressure

The ISP probe was connected to a Codman intracranial pressure box linked via an ML221 amplifier connected to a PowerLab running LabChart 8 (ADInstruments, Oxford, UK). Blood pressure was recorded from a radial artery catheter connected to the Philips IntelliVue MX800 bedside monitoring system (Philips, Guildford, UK) and, in turn, connected to the PowerLab system. ISP and arterial blood pressure (ABP) signals were sampled at 1 kHz, and patients were monitored for up to 7 days. Figure 1 shows the setup. Data were analyzed using LabChart 8 (ADInstruments) and ICM + (University of Cambridge, Cambridge, UK; available at: www.neurosurg.cam.ac.uk/icmplus). ISP and ABP were used to compute SCPP = MAP − ISP.

### Neuro-ICU Clinical Management

Patients were transferred to the neuro-ICU following surgery and remained there for the duration of spinal cord monitoring and clinical need. Patients were nursed on pressure-relieving mattresses on their side, when possible, to take pressure off the laminectomy site. Antibiotic prophylaxis was given for 48 h after surgery with vancomycin and gentamicin, which is the standard regimen in our hospital for instrumented spinal fusion. Subcutaneous prophylactic low-molecular-weight heparin was started at 24 h post surgery (held for 12 h pre and post probe removal). Mechanical (compression stockings, intermittent calf compression) thromboprophylaxis was used throughout. Ventilatory support was managed by the neuro-ICU team. MAP targets were at the discretion of the neuro-ICU (unrelated to ISP or SCPP), except for when the SCPP was altered to produce low and high SCPP for ARM measurements. Ionotropic support was provided with intravenous noradrenaline through a central venous line or, while central access was being established, with intravenous metaraminol via a peripheral venous line. All patients were reviewed and examined daily by the research team, and any complications or concerns were recorded and actioned immediately. Per neuro-ICU protocols, daily blood tests were performed, including full blood cell count, renal and liver profile, cardiac enzymes, C-reactive protein, and clotting function.

### ARM

All patients underwent ARM in the neuro-ICU over a wide range of SCPPs in the week following surgery. The duration of ARM was determined by how long the patient was able to tolerate the intervention and whether the clinical condition of the patient was appropriate for this testing. AP was sampled at 1 kHz with a 4-channel water perfused manometer (Ardmore, Middleton-on-Sea, UK) leveled at the anal verge (Fig. 1). The pressure monitor was connected to the Philips IntelliVue MX800 bedside monitoring system (Philips), which, in turn, was connected to the PowerLab system. AP was monitored at rest, during cough, during squeeze, and during the RAIR and balloon threshold testing. Resting and maximal AP was taken as the mean of the four channels, excluding rectal recordings. During cough, we noted the maximum AP and the increment from resting AP (ΔAP). During prolonged (10 s) anal squeeze, we noted the endurance time (duration AP as greater than 50% of maximum) and the increase in AP from resting pressure (ΔAP). The RAIR was performed by rapid inflation to 50 ml and deflation of the rectal balloon and was assessed for baseline AP, latency, maximal amplitude, amplitude reduction, recovery time, and duration. Rectal sensation threshold tests were estimated by filling the rectal balloon at 50-ml increments (to a maximum of 500 ml) and by asking the patient to comment on first sensation, first desire to defecate, and urgency to defecate. For cough, squeeze, and RAIR, repeated assessments were performed in each patient at low and high SCPP.

### Variables Assessed and Bias

We investigated the relation between baseline AP and SCPP, the characteristics of the RAIR at low versus high SCPP, the effect of cough on AP at low versus high SCPP, and the effect of voluntary anal squeeze on AP at low versus high SCPP. For baseline AP, we used data from the entire monitoring period at 1 Hz. The average hours of monitoring for each patient were 1.9 (range 0.4–12). To assess RAIR, cough, and rectal sensation (elicited by inflating a rectal balloon), SCPP was manipulated using noradrenaline. Each patient had a period when the dose of noradrenaline was reduced and a period when the noradrenaline was increased. Each figure highlights the average SCPP for each patient at their low and high SCPP periods of monitoring. We also asked the patients to strain at different SCPPs. To minimize bias, the ARM data were obtained without knowledge of the SCPP.

### Statistics

Plots of ARM parameters (resting AP, prolonged squeeze endurance time, prolonged squeeze ΔAP vs. SCPP) were fitted with the best-fit quadratic or linear regression curve, chosen to minimize the Akaike information criterion. Adjusted coefficients of determination (*Ȓ*^2^) were computed using MyCurveFit (https://mycurvefit.com), with *Ȓ*^2^ values greater than 0.5 being considered a strong correlation. The relation between mean values of components of RAIR (baseline AP, recovery time, percentage of amplitude reduction) and cough (maximum AP, mean ΔAP) at high and low SCPP were compared using Student’s *t* test. The correlation between AIS grade or SCPP and anal sphincter outcome was assessed using Kendall’s tau-b correlation coefficient. Analysis was done using XLSTAT Biomed v2018.1 for Mac (Addinsoft, Paris, France). Statistical significance was taken as *P* values of less than 0.05.

## Results

### Patient Demographics

Data were obtained from 14 patients aged 22–67 years (mean 47.4): 13 men and 1 woman. Most (64.3%) patients had complete (AIS grade A) injuries, and most (64.3%) had cervical injuries. All patients had posterior surgical bony decompression, including laminectomy and fusion, with 21.4% also requiring anterior stabilization. The mean follow-up time was 8.1 months (range 2–19). At follow-up, most (71.4%) patients had improved by one of more AIS grades, 21.4% remained the same, and 7.2% deteriorated by one AIS grade. These demographics are summarized in Table 1.

**Table 1:** Patient details

Pt. No.	Age (y)	Sex	Injury level	AIS	Injury to surgery (h)	Surgery	Follow-up (m)	Follow-up AIS
71	27	M	L1	C	41	Post + Lami	6	D
72	50	M	C5	B	14	Post + Lami	19	B
73	47	M	T8	A	23	Post + Lami	12	B
74	57	M	C4	A	35	Post + Lami	8	A
75	66	M	C4	A	40	Post + Lami	7	A
76	46	M	T12	A	18	Post + Lami	9	C
77	52	M	C5	A	40	Ant + Post + Lami	8	B
78	26	M	C6	A	39	Ant + Post + Lami	12	B
79	67	F	C2	B	69	Post + Lami	2	A
80	55	M	T7	A	45	Post + Lami	14	C
81	54	M	C4	C	69	Post + Lami	6	D
82	44	M	C7	A	32	Post + Lami	4	A
83	51	M	T7	A	50	Post + Lami	5	A
84	22	M	C6	C	70	Ant + Post + Lami	2	D

	Mean (SEM)	M:F (*n*)	C:T:L (*n*)	A:B:C:D (*n*)	Mean (SEM)	Ant, Post, Lami/14 (*n*)	Mean (SEM)	A:B:C:D (*n*)
All	47.4 (3.7)	13:1	9:4:1	9:2:3:0	41.8 (4.8)	3, 14, 14/14	8.1 (1.3)	5:4:2:3

AIS, American Spinal Injuryies Association Impairment Scale; Ant, anterior; C, cervical; F, female; h, hours; L, lumbar; Lami, laminectomy; m, months; M, male; No., number, Post, posterior, Pt., patient; Post, posterior; SEM, standard error of the mean; Pt., patient; T, thoracic; y, years

### ARM

We monitored resting AP for an average of 1.9 h per patient (range 0.4–12) over an average of 2.1 days per patient (range 1–4). Per patient, the average number of coughs was 11.8 (range 1–36), the average number of voluntary squeezes was 4.9 (range 0–13), and the average number of RAIRs was 10.9 (range 0–19). In some patients, the cough and squeeze tests could not be performed because of sedation used for respiratory support. At follow-up, the NBD score classified 21.4% of patients as having minor, 28.6% moderate, and 50.0% severe bowel problems. For the SCIM III bowel scores, 7.1% of patients scored 0, 50.0% scored 5, 28.6% scored 8, and 14.3% scored 10. Details are in Table 2.

**Table 2:** Summary of anorectal manometry investigations

Pt. No.	Monitoring duration (h)	Monitoring span (d)	Mean resting AP (cmH_2_O)	No. of coughs (*n*)	No. of strains (*n*)	No. of RAIRs (*n*)	SCIM III bowel score^a^	NBD score^b^
71	12.0	1	30.2	8	0	0	8	15
72	4.0	3	64.8	1	0	3	5	18
73	1.4	4	42.7	36	0	8	0	21
74	1.7	3	32.0	12	0	16	5	11
75	0.8	3	59.5	23	13	15	5	19
76	0.5	2	54.3	16	10	11	8	28
77	0.9	2	42.2	13	12	9	5	15
78	0.8	2	47.1	1	1	15	10	2
79	0.4	1	45.4	1	1	19	5	19
80	0.7	2	40.3	8	7	9	8	10
81	0.8	2	41.3	10	7	13	8	2
82	0.9	2	34.1	12	7	12	5	10
83	0.4	1	48.3	7	8	11	5	13
84	1.1	2	37.8	17	2	12	10	0

	Mean SEM	Mean SEM	Mean SEM	Mean SEM	Mean SEM	Mean SEM	Mean SEM	Mean SEM
Mean (SEM)	1.9 (0.8)	2.1 (0.2)	44.3 (2.7)	11.8 (2.5)	4.9 (1.3)	10.9 (1.3)	6.2 (0.7)	13.1 (2.1)

AP, anal pressure; d, days; h, hours; NBD, neurogenic bowel dysfunction; No., number; Pt., patient; RAIR, recto-anal inhibitory reflex; SCIM III, Spinal Cord Independence Measure III; SEM, standard error of the mean; SCIM III, Spinal Cord Independence Measure

^a^SCIM III: 0, irregular timing or very low frequency (less than once in 3 days) of bowel movements; 5, regular timing, but requires assistance (e.g., for applying suppositories)—rare accidents (less than twice a month); 8, regular bowel movements, without assistance—rare accidents (less than twice a month); 10, regular bowel movements, without assistance—no accidents

^b^Bowel dysfunction: 0–6, very minor; 7–9, minor; 10–13, moderate; 14+, severe

### Complications

There were no complications associated with ARM. Seven patients had asymptomatic pseudo-meningoceles on the postoperative magnetic resonance imaging scan, five were treated for chest sepsis, one had a pulmonary embolism, and one developed a sacral pressure sore.

### Resting AP

**Fig. 2:**
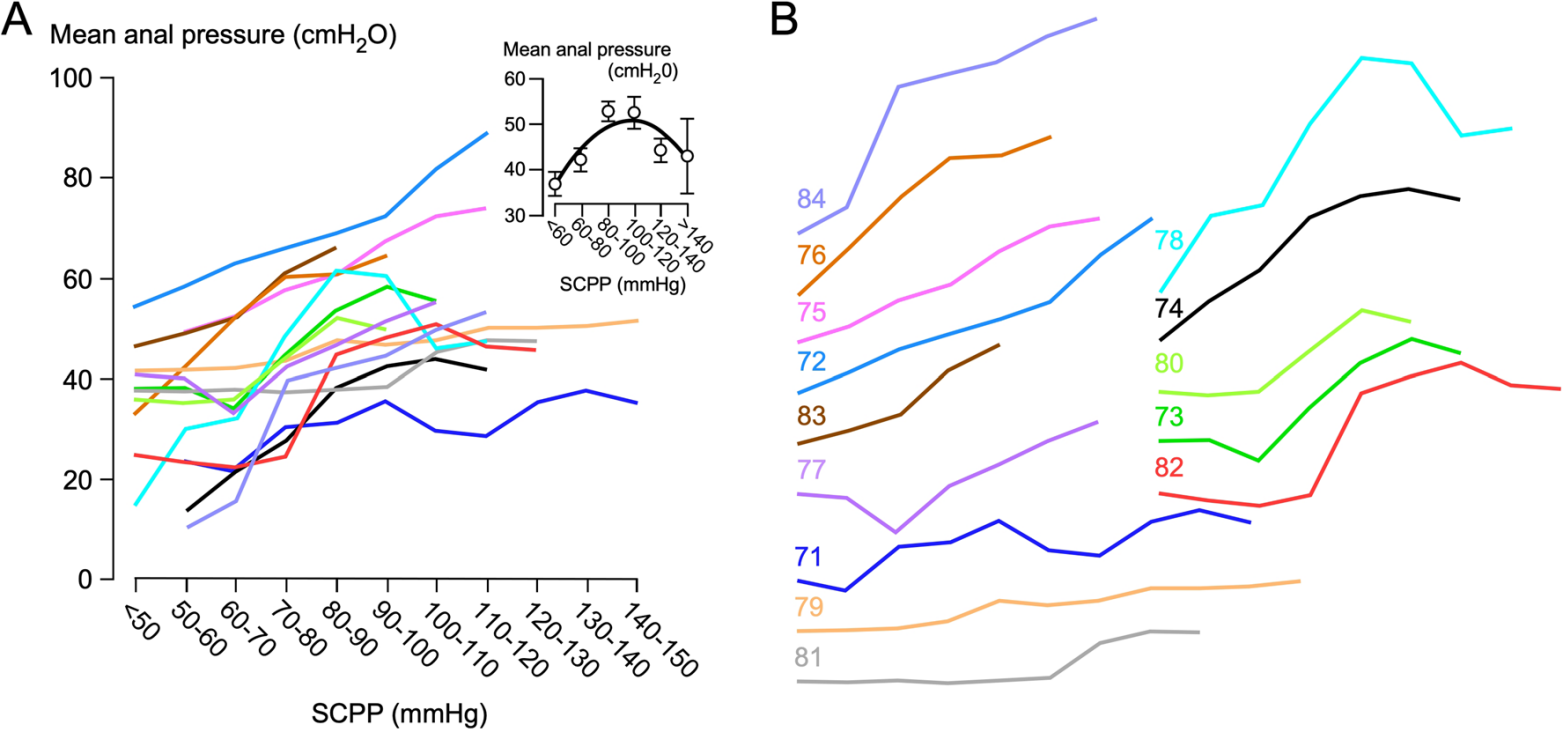
Correlation between SCPP and AP. **a** AP versus SCPP for each of the 14 patients. AP averaged for all patients versus SCPP (inset). Mean ± standard error with best-fit quadratic, *Ȓ*^2^ = 0.82. **b** Individual curves from **a** spread out. Colors correspond to patients as shown. AP anal pressure, SCPP spinal cord perfusion pressure

Altogether, we collected 26.3 h of resting AP data, i.e., 1.9 ± 0.8 h per patient. Average resting AP was 44.3 cmH_2_O, which is considerably lower than the average for healthy male patients, previously reported at 99 cmH_2_O [23]. For most patients, higher SCPP was associated with higher resting AP, but when the data were grouped, mean AP versus SCPP showed an inverted U-shaped relation (*Ȓ*^2^ = 0.82; Fig. 2). Maximal AP showed a similar correlation with SCPP as mean AP (Supplementary Fig. 1). In the seven patients who had intraoperative monitoring, there was no significant change in resting AP pre versus post bony spinal cord decompression (Supplementary Fig. 2). There was no correlation between the average or maximum resting AP and ISP (Supplementary Figs. 3 and 4). Also, there was no correlation between the dose of noradrenaline and the average AP, thus making it unlikely that the relation between SCPP and AP is due to a direct effect of noradrenaline on the anal sphincter (Supplementary Fig. 5). Because low resting AP is associated with incontinence, our findings suggest improved fecal continence at higher SCPP.

### RAIR

**Fig. 3:**
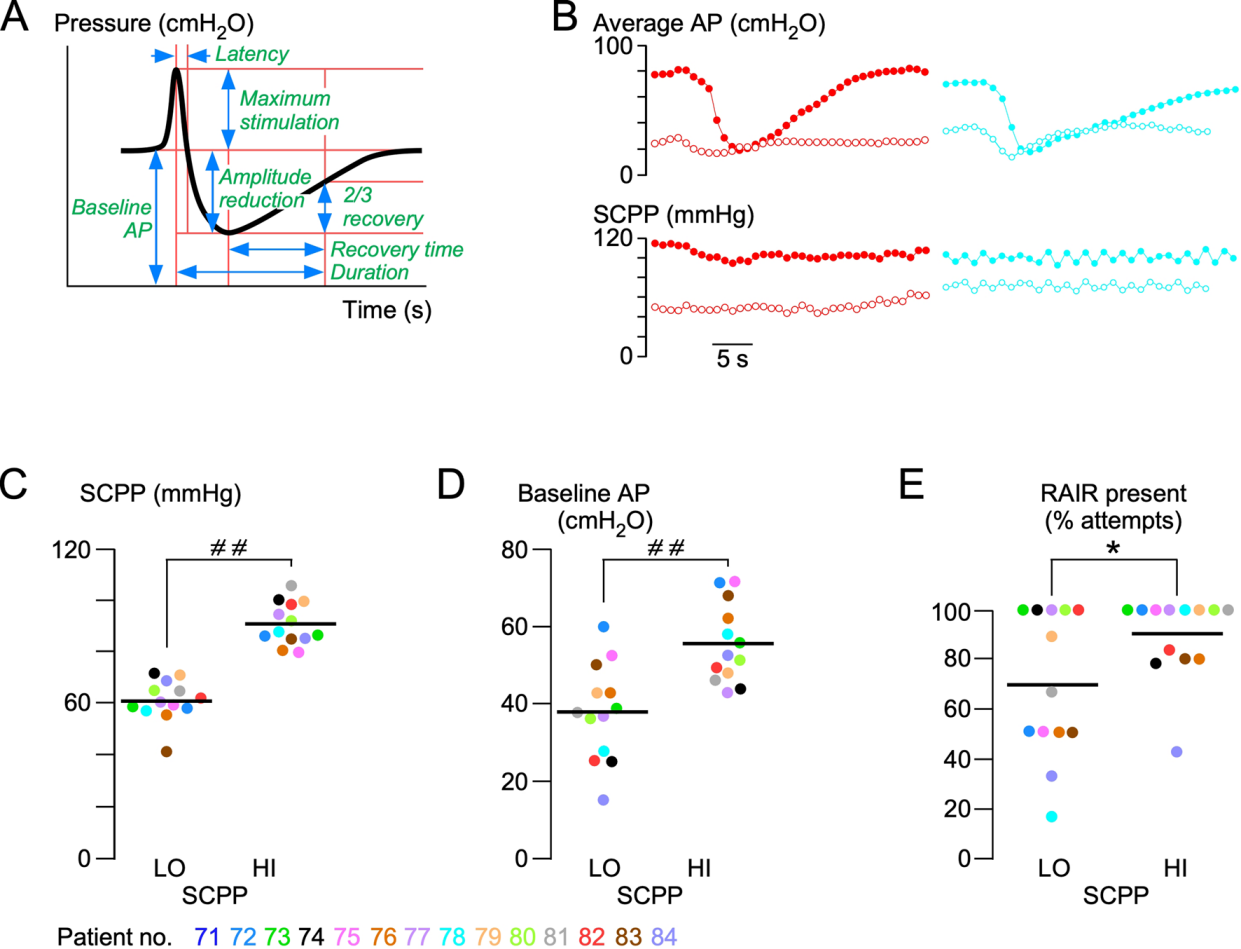
Effect of SCPP on the RAIR. **a** Schematic showing characteristics of the RAIR signal. **b** Typical RAIR signals from patients 82 (left) and 78 (right) at low (open circles) and high (solid circles) SCPPs. **c** Mean high (HI) and mean low (LO) SCPPs corresponding to the RAIR measurements for each patient. Plots showing individual patient values (points) and means (lines) at HI versus LO SCPP of mean baseline AP (**d**), recovery time (**e**), and percentage amplitude reduction of the RAIRs (**f**). Color codes for patients 71–84. *P < 0.05, ^# #^*P* < 0.0001. *AP* anal pressure, *RAIR* recto-anal inhibitory reflex, *SCPP* spinal cord perfusion pressure

The RAIR was observed in all 13 patients in whom it was tested but not in all traces from each patient (Fig. 3). The SCPP had a major impact on the RAIR: compared with low SCPP (60.3 mmHg), high SCPP (90.0 mmHg) was associated with a higher baseline AP (55.6 vs. 35.2 cmH_2_O, respectively), and the RAIR was more likely to be present at the higher SCPP (89.7 vs. 69.5% of attempts). When considering only the AP traces with the RAIR present, there was no difference in the RAIR characteristics (excitation latency, duration of reflex, percentage of amplitude reduction, recovery time) at low versus high SCPP. The RAIR is an essential reflex for the voluntary control of defecation because it enables the rectum to discriminate between gas, liquid, and solid contents [24, 25]. Therefore, the more frequent presence of the RAIR at higher SCPP suggests an increased continence at higher SCPP.

### AP During Cough

**Fig. 4:**
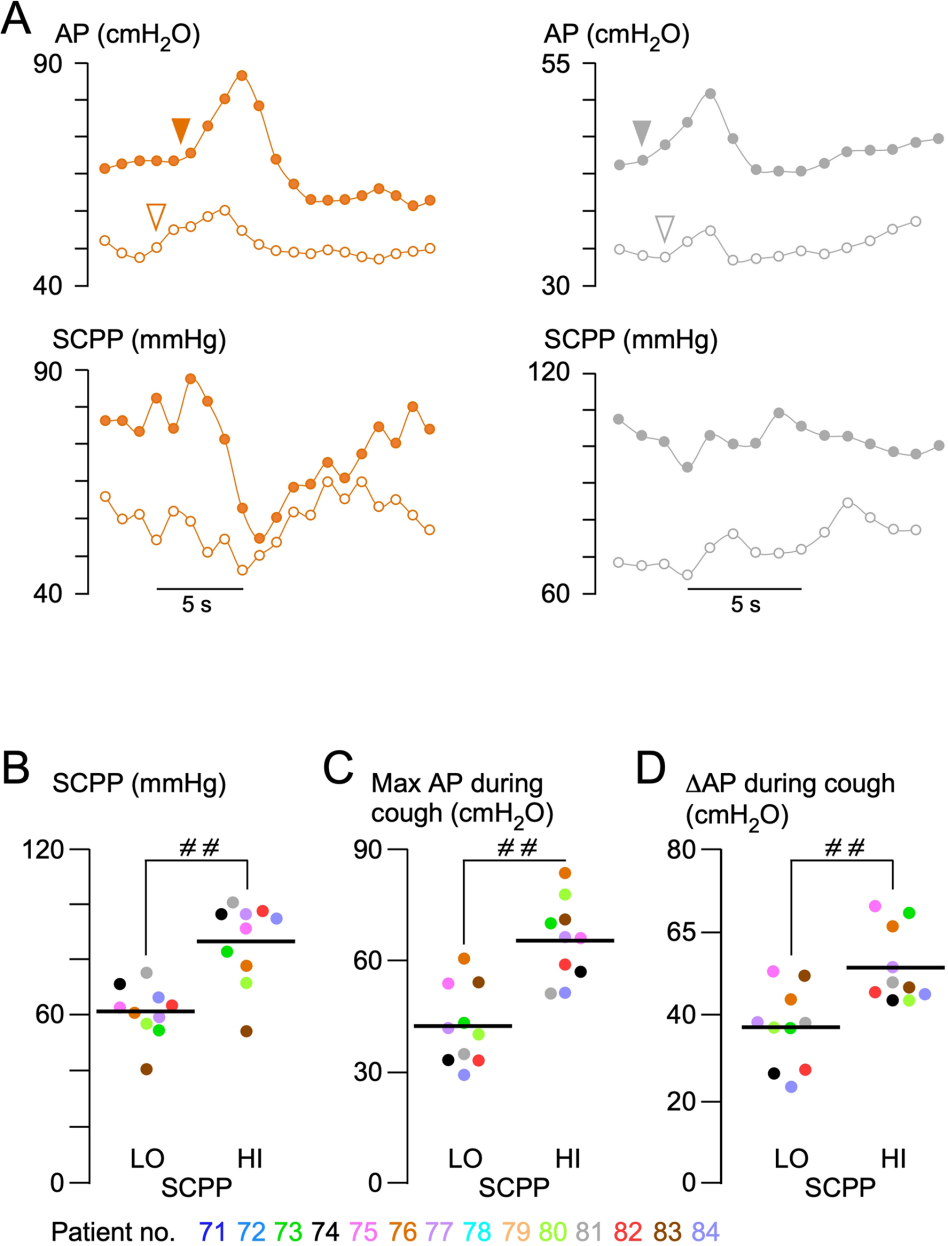
Effect of cough on AP. **a** AP changes during cough for patients 6 (left) and 11 (right) at low (open circles) and high (solid circles) SCPPs. Plots showing individual patient values (points) and means (lines) at high (HI) versus low (LO) SCPP of mean SCPP (**b**), mean maximum AP (**c**), and mean change in AP during cough (**d**). Color codes for patients 71–84. ^# #^*P* < 0.0001. *AP* anal pressure, *SCPP* spinal cord perfusion pressure

Cough tests were performed in all patients. In 11 patients, cough was assessed at high (81.6 mmHg) and low (61.4 mmHg) SCPP (Fig. 4). At high SCPP, the maximum AP during cough was significantly higher than at low SCPP by over 20.0 cmH_2_O (*P* < 0.0001) on average. The increase in AP compared with resting pressure prior to the cough was also higher at high versus low SCPP by approximately 14.7 cmH_2_O (*P* < 0.0001) on average. Because episodes of incontinence are more likely during cough, our data indicate that anal sphincter continence is improved at higher SCPP.

### AP During Voluntary Squeeze

**Fig. 5:**
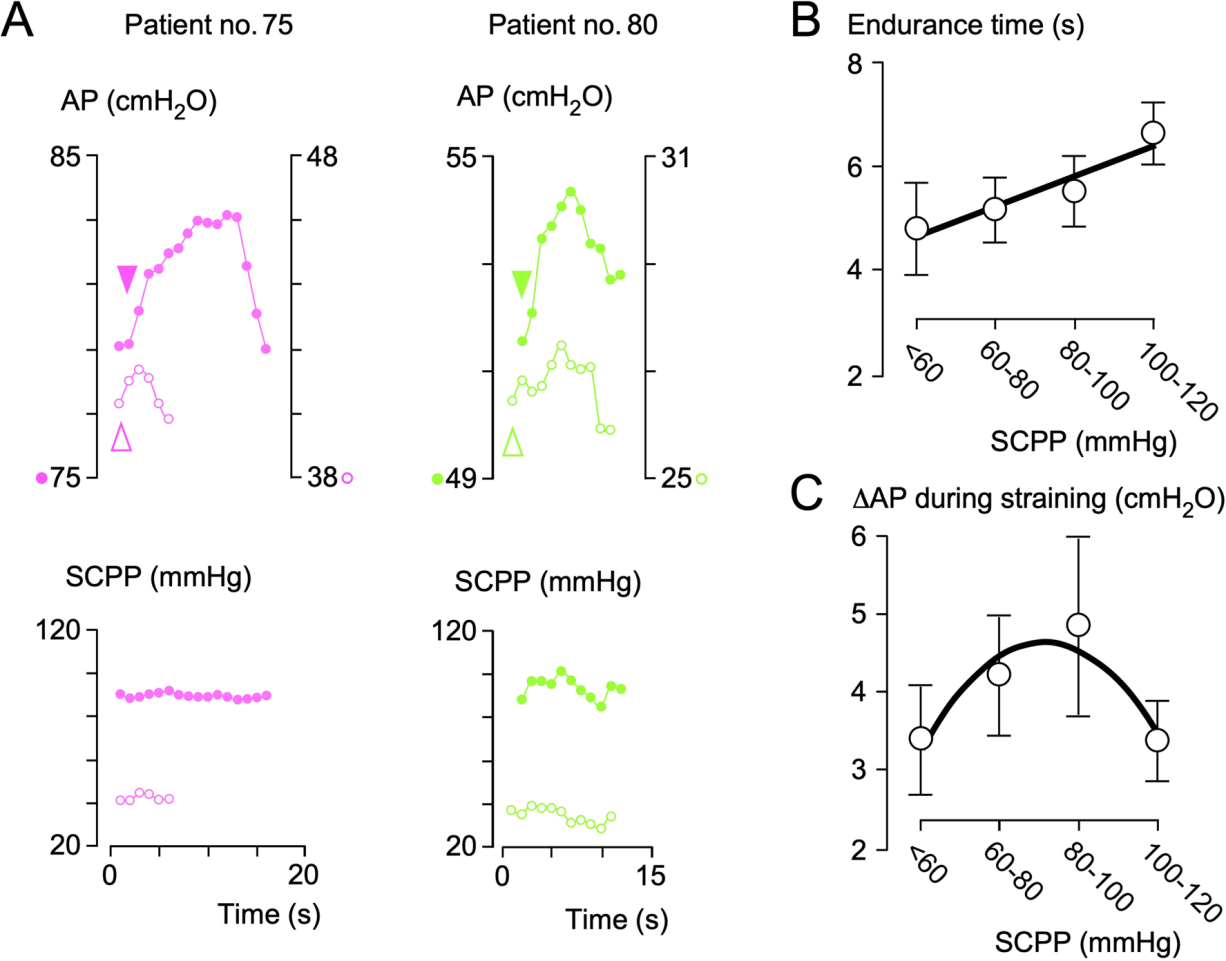
Effect of prolonged squeeze on AP. **a** AP changes during squeeze for patients 5 (left) and 10 (right) at low (open circles) versus high (solid circles) SCPPs. Plots of endurance time (**b**) and change in AP (**c**) during straining. *N* = 10, mean ± standard error. Best-fit straight line (**a**, *Ȓ*^2^ = 0.93) and quadratic (**b**, *Ȓ*^2^ = 0.87). *AP* anal pressure, *SCPP* spinal cord perfusion pressure

Increase in AP during squeeze was significantly reduced in our patients compared with reported normal male controls (mean endurance time 5.3 vs. 16 s, mean ΔAP 3.7 vs. 195 cmH_2_O) [23]. Figure 5 shows that the sphincter response has a longer endurance at a higher SCPP. An inverted U-shaped curve fit the relationship between ΔAP and SCPP, with the sphincter showing a stronger response at a SCPP of approximately 80 mmHg and a weaker response at hypoperfusion and hyperperfusion (*Ȓ*^2^ = 0.87).

### Rectal Sensation

Threshold tests for rectal sensation were performed in 10 of 14 patients at high and low SCPP. The other four patients were sedated for ventilatory reasons. Six of ten patients reported rectal sensation during the tests (4 AIS grade A, 2 AIS grade C). At follow-up, all six patients who had rectal sensation at presentation had the same or improved AIS grade. The four patients lacking rectal sensation at presentation all had AIS grade A and remained so. The probability of experiencing rectal sensation, first urge to defaecate, or maximum tolerance versus volume of the balloon was the same at high and low SCPP (Supplementary Fig. 6).

### Role of MAP

We tested whether MAP could be used as a surrogate for SCPP (Supplementary Fig. 7). In all patients, there was a strong positive correlation between MAP and SCPP but with wide interpatient variability. As MAP increases, mean AP increases and then plateaus, but as SCPP increases, mean AP rises and then falls (Fig. 2). For prolonged squeeze (Fig. 5), there was a strong linear correlation between endurance time and SCPP and a strong correlation between ΔAP and SCPP but no correlation between endurance time and MAP (*Ȓ*^2^ = 0.07) and only a weak correlation between ΔAP and MAP (*Ȓ*^2^ = 0.47). We conclude that MAP is not a good surrogate for SCPP.

### Long-Term Outcomes

**Fig. 6:**
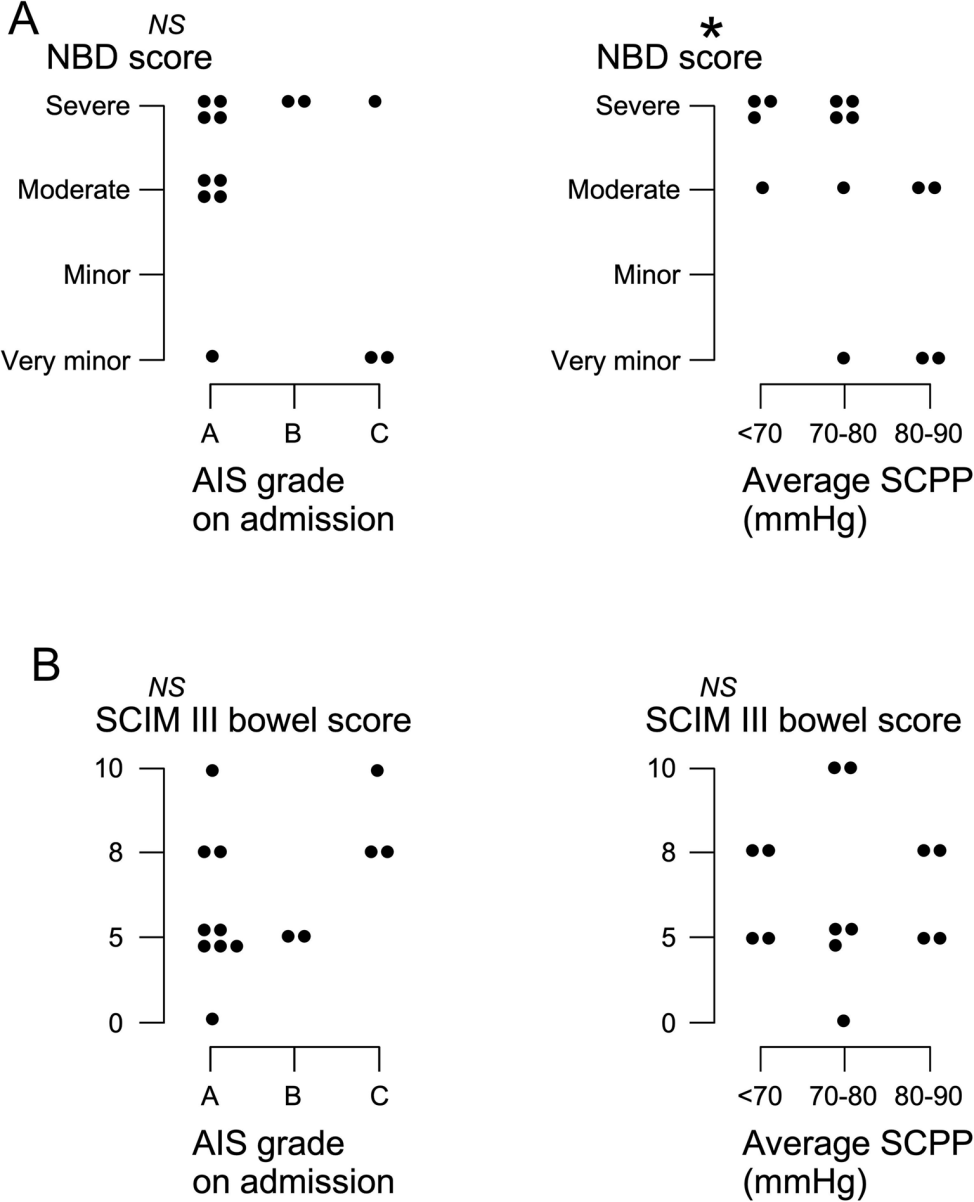
Bowel function at follow-up. **a** NBD scores versus (left) AIS grade and (right) versus average SCPP on admission. **b** SCIM III bowel scores versus (left) AIS grade and (right) versus average SCPP on admission. *P < 0.05. *AIS* American Spinal Injury Association Impairment Scale, *NBD* Neurogenic Bowel Dysfunction, *NS* not significant, *SCIM III* Spinal Cord Independence Measure III, *SCPP* spinal cord perfusion pressure

There was no correlation between AIS grade on admission and anal sphincter function at follow-up (NBD and SCIM III bowel scores). There was a significant correlation between the average SCPP on admission and anal sphincter function at follow-up assessed with the NBD score (*P* < 0.05) but not with the SCIM III bowel score (see Fig. 6).

## Discussion

We showed that TSCI causes severe disruption of anal sphincter function. Our key finding is that intervention to increase the SCPP improves several components of anal continence, including resting AP, RAIR, AP during cough, and AP during squeeze. We also showed evidence that too high of SCPP may worsen anal sphincter continence. Being able to improve anal sphincter function early by optimizing the SCPP is a compelling prospect.

The reported variability in ARM findings and its dependence on physiological changes at the injury site (e.g., change in SCPP) suggest that interventions to optimize injury-site physiology may improve anal function. This is further supported by the finding that the average SCPP on admission correlated with the NBD score at follow-up. Ultimately, a randomized controlled trial is required to definitively determine whether intervening to optimize the SCPP improves outcome. Intervention to increase SCPP (SCPP = MAP − ISP) may be achieved by increasing the MAP using inotropes [6] or by reducing the ISP using expansion duroplasty [26]. The finding that overincreasing the SCPP may worsen anal sphincter function suggests that not only hypoperfusion but also hyperperfusion at the injury site is detrimental. The notion that injury-site hyperperfusion is detrimental after TSCI is also supported by our earlier studies that used injury-site autoregulation [6], injury-site blood flow [27], limb power [13], and urinary bladder function [14] as outcomes.

ARM offers mechanistic insight into the impact of TSCI on anal sphincter function. We found that in the acute phase, TSCI causes relaxation of the anal sphincter. The anal sphincter no longer contracts effectively in response to cough or voluntary squeeze, and the RAIR is not always present. As a result of these disruptions, fecal continence can no longer be maintained.

We did not detect an effect of increasing SCPP on the rectal volumes required to elicit sensations (first sensation, first urge to defecate, maximum tolerance). The data show a trend, i.e., increasing SCPP reduces the rectal volume required to first produce desire to defecate or to first produce maximum tolerance, although these findings did not reach significance. The small number of patients in our study may be the limiting factor here, and it is possible that increasing the number of patients may yield significance.

Our study has limitations. First, some patients were sedated because they required ventilation, which prohibited them from participating in the squeeze and sensory threshold tests. Second, some patients received noradrenaline infusions, raising the possibility of a direct effect of noradrenaline on the anal sphincter. Our finding that the dose of noradrenaline does not correlate with resting AP suggests that a direct effect of noradrenaline on the anal sphincter is unlikely. Third, because of the acute intensive care unit setting, we were unable to perform additional studies, e.g., high resolution ARM or anal ultrasound. Fourth, the number of patients in our study is relatively small because of the difficulty in performing multiple ARM assessments in patients with acute injuries in the neuro-ICU. Importantly, no patient experienced complications directly related to ARM testing. The outcomes of patients with TSCI who undergo monitoring from the injury site are comparable with the outcomes of patients who were not monitored, as previously reported [10].

The finding that optimizing SCPP is associated with improved anal sphincter function is in line with our earlier findings that optimizing SCPP is associated with improved limb motor score [6, 13], sensory level [28], and urinary bladder function [14]. Our analysis suggests that using MAP as a surrogate for SCPP is inadequate because patients who have the same MAP and different ISP will have different SCPP. A randomized controlled trial, Duroplasty for Injured Spinal Cord with Uncontrolled Swelling, funded by the UK National Institute for Health Research (NIHR130048), has been set up to determine if expansion duroplasty improves neurological outcomes, including anal sphincter function, compared with standard treatment for patients with acute, severe cervical TSCI. The trial includes optional monitoring from the injury site.

## Conclusions

Our ARM findings suggest that individualized therapy in acute TSCI, through monitoring and optimizing spinal cord physiology and metabolism, may improve not only limb motor function, sensation, and urinary function but also anal sphincter function.

## Supplementary Information

Below is the link to the electronic supplementary material.

Supplementary file1 (PDF 1329 kb)

## Abbreviations

ABP: Arterial blood pressure
AP: Anal pressure
ARM: Anorectal manometry
ISCoPE: Injured spinal cord pressure evaluation
ISP: Intra cranial pressure
MAP: Mean arterial pressure
NBD: Neurogenic bowel dysfunction
NICU: Neuro-intensive care unit
RAIR: Recto-anal inhibitory reflex
SCIM: Spinal cord independence measure
SCPP: Spinal cord perfusion pressure

## Clinical trial registration

www.ClinicalTrials.gov: NCT02721615.
