# Supplementary material for: Spinal Cord Perfusion Pressure Correlates with Anal Sphincter Function in a Cohort of Patients with Acute, Severe Traumatic Spinal Cord Injuries
Source: Neurocrit Care. 2021 Jun 7;35(3):794–805. doi: 10.1007/s12028-021-01232-1 (PMC8692299; doi:10.1007/s12028-021-01232-1)
Supplement: Supplementary file 1 — Supplementary file1 (PDF 1329 kb) [file 12028_2021_1232_MOESM1_ESM.pdf]

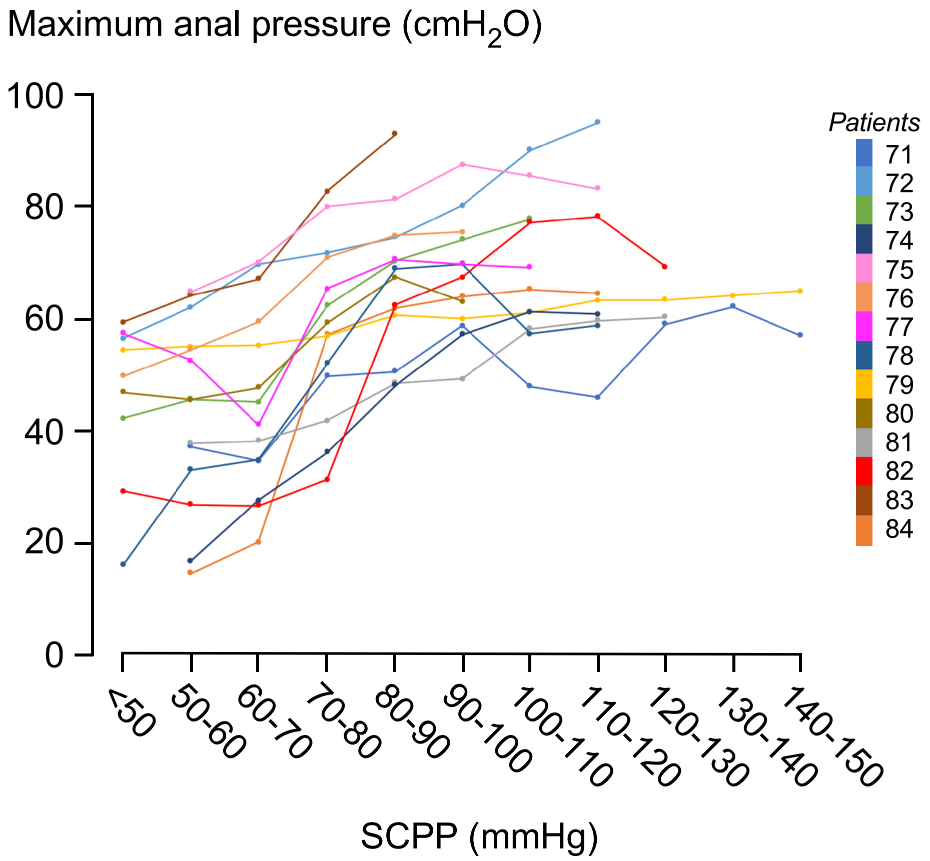

S-Fig. 1. Plot of maximum anal pressure versus SCPP for individual patients. Each patient is a different colour.

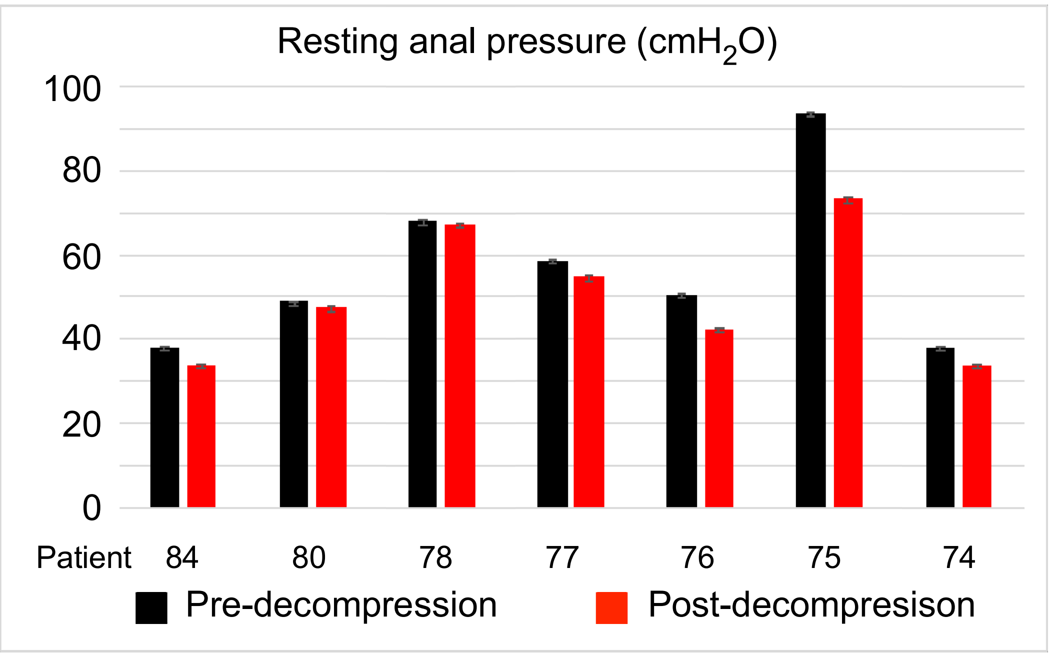

S-Fig. 2. Effect of surgical decompression on mean resting anal pressure in seven patients.

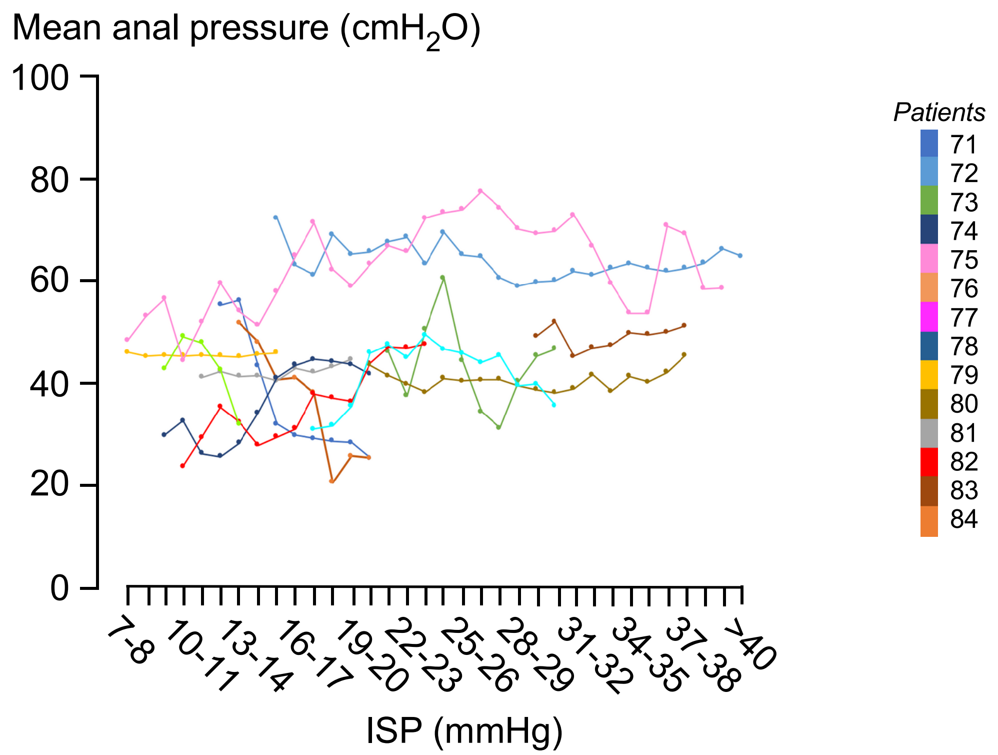

S-Fig. 3. Plot of mean anal pressure versus ISP for individual patients. Each patient is a different colour.

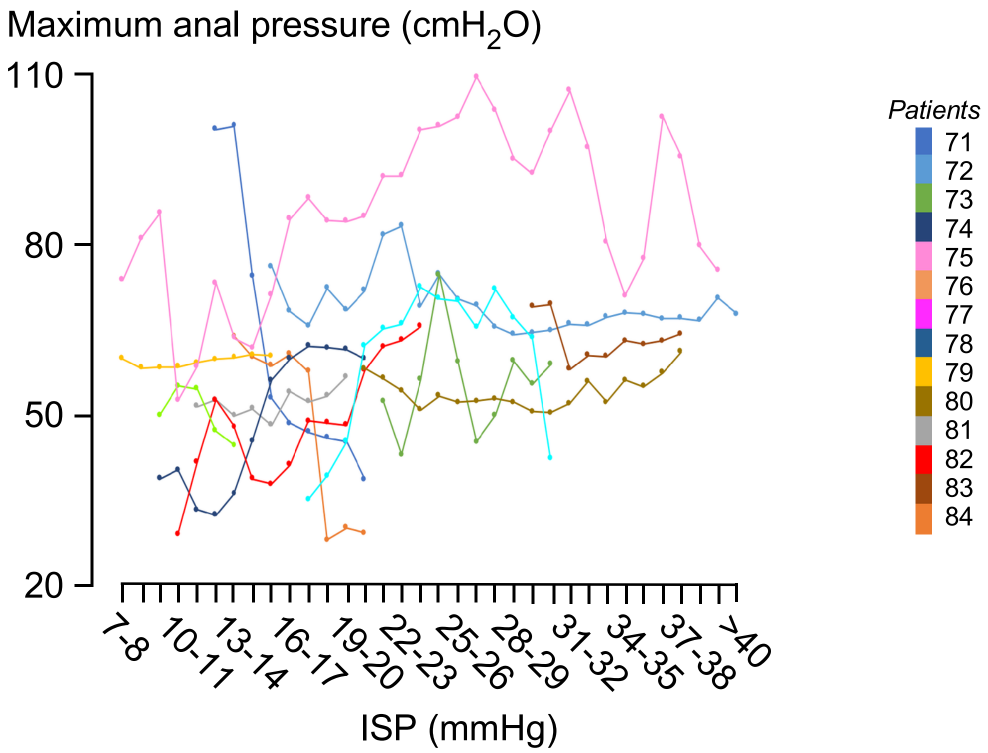

S-Fig. 4. Plot of maximum anal pressure versus ISP for individual patients. Each patient is a different colour.

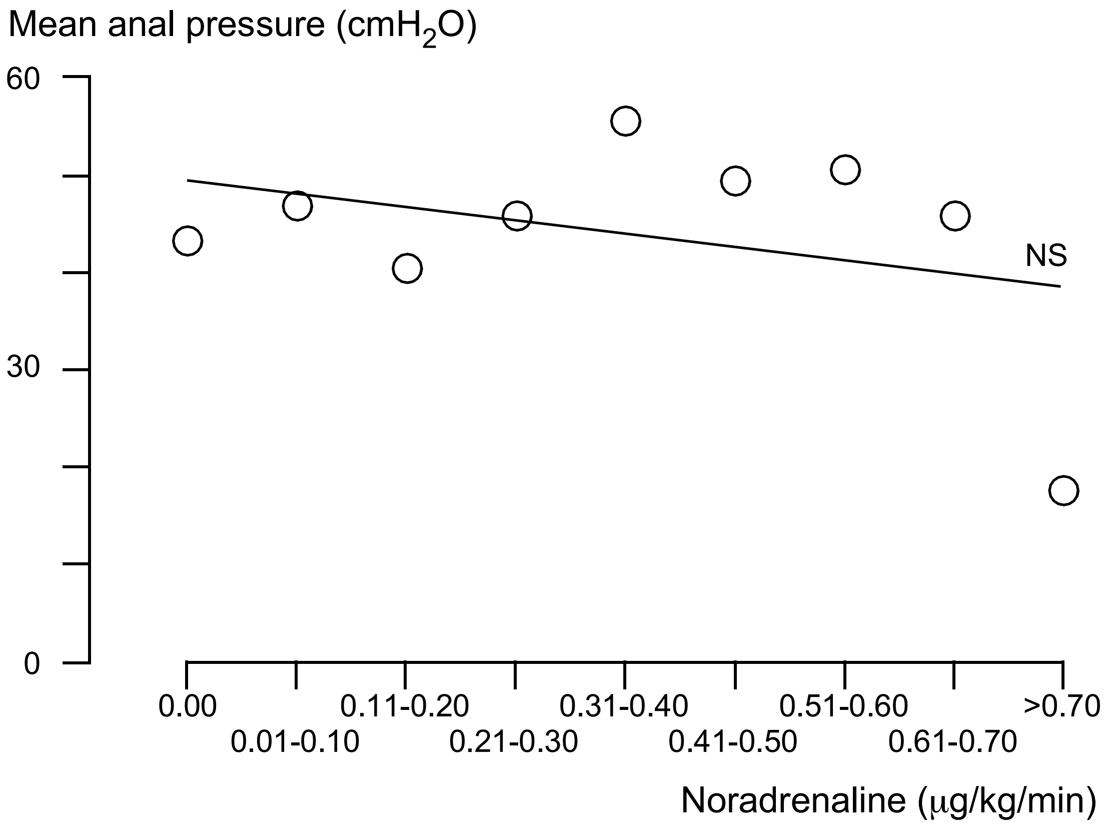

S-Fig. 5. Relation between dose of noradrenaline and average anal pressure. Data from 14 patients, 429,908 data points. $R^2 = 0.12$.

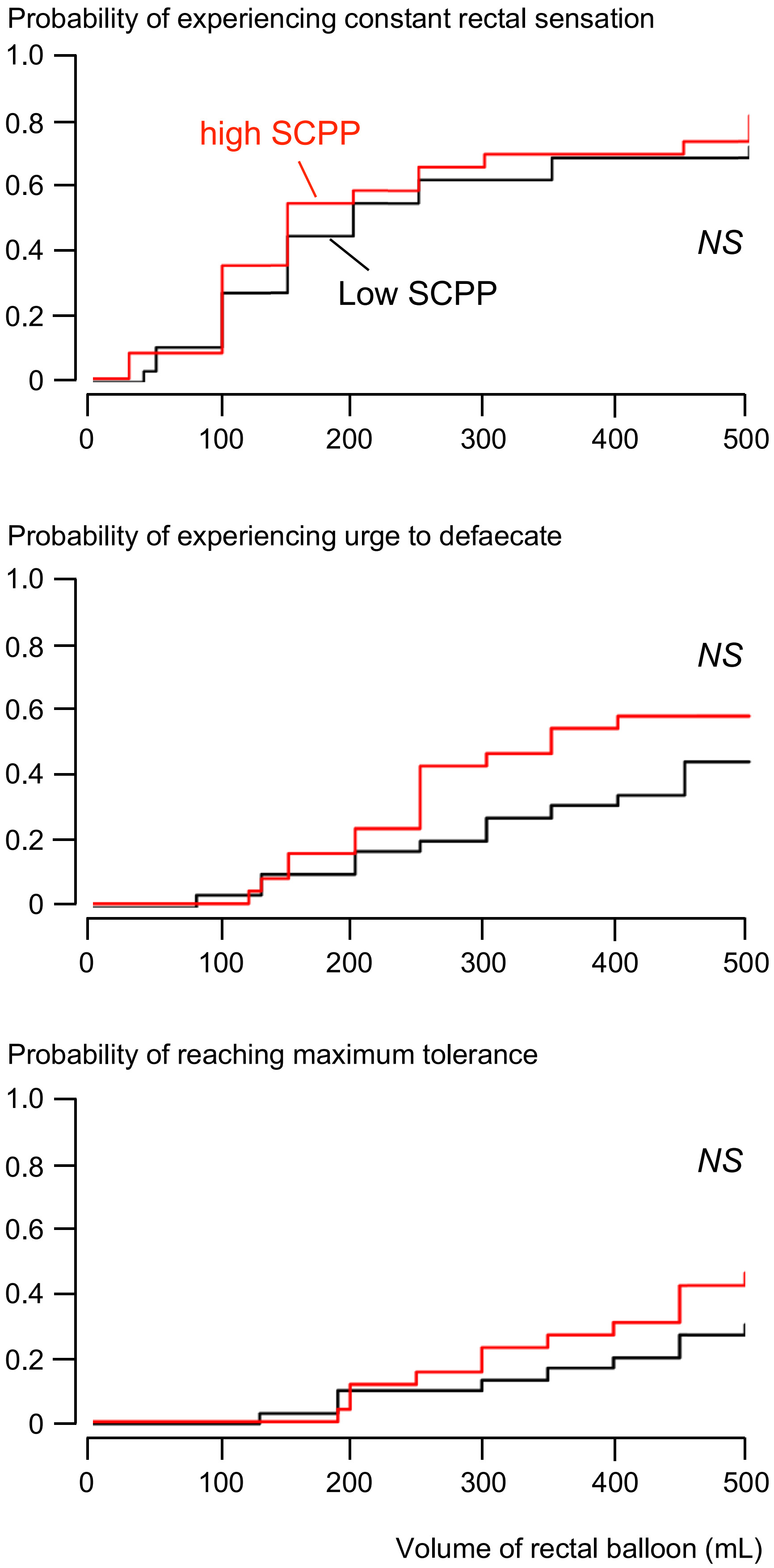

S-Fig. 6. Effect of SCPP on rectal sensations. A. Probability of not experiencing first constant rectal sensation vs. volume of rectal balloon. B. Probability of not experiencing first urge to defaecate vs. volume of rectal balloon. C. Probability of not reaching the maximum tolerance vs. volume of rectal balloon. Plots at high (red, 91.7 +/- 1.7 mmHg) and low (black, 62.2 +/- 1.4 mmHg) SCPP. *NS*, not significant.

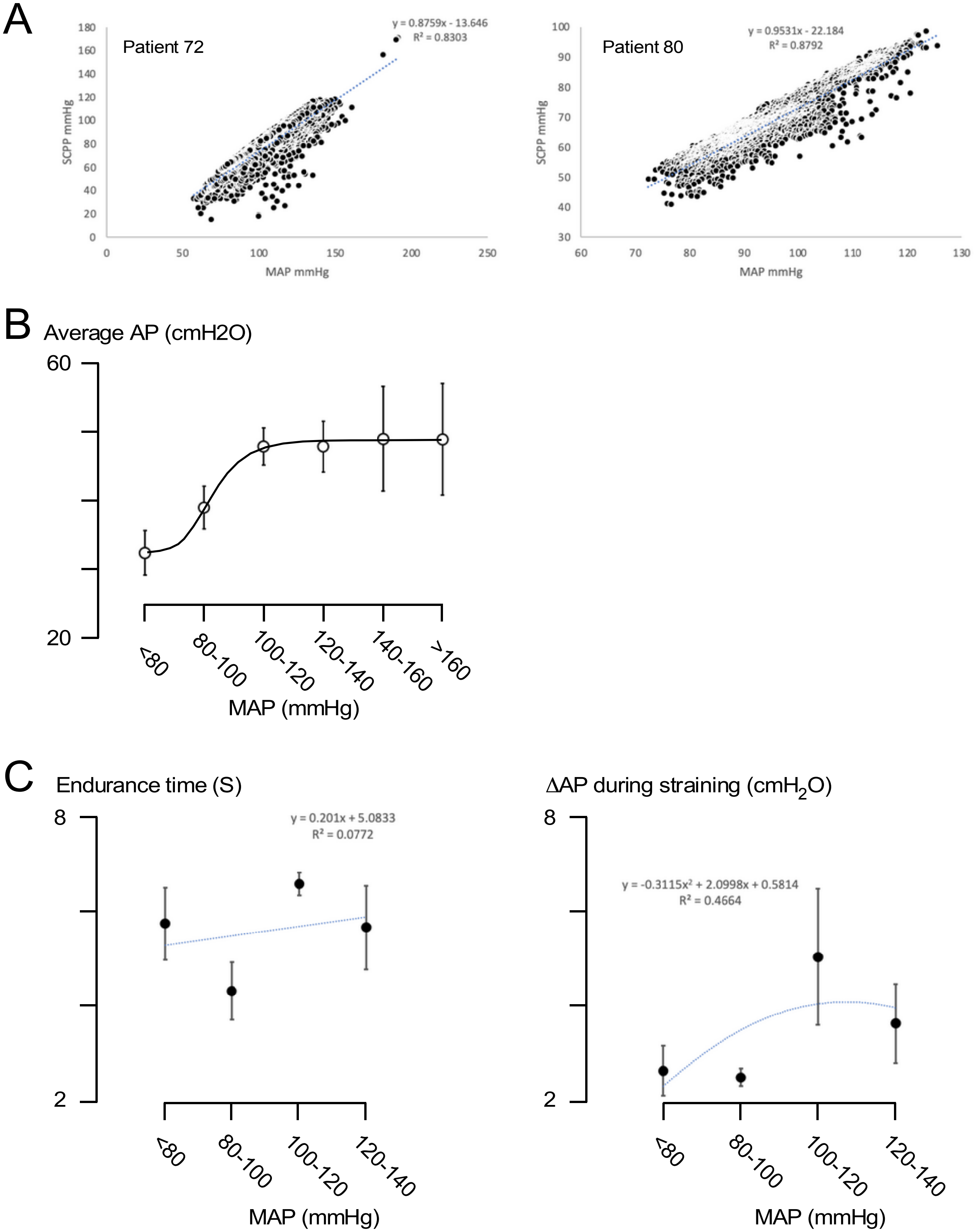

S-Fig. 7. MAP is not a good surrogate for SCPP. A. Plots of SCPP vs. MAP for patients 72 and 80. B. Plot of mean AP vs. MAP. C. Plots of Endurance time and ΔAP during straining vs. MAP. Mean +/- sem (B, C, D). Best fit linear (A, C left), quadratic (C right) and sigmoid (B).
